# Robotergestützte Rektumresektionen – Scoping-Review für Klasse-1a-Evidenz und retrospektive Analyse klinikinterner Daten

**DOI:** 10.1007/s00104-022-01774-w

**Published:** 2022-11-30

**Authors:** Maria A. Willis, Sophia L. V. Soltau, Cornelius van Beekum, Nils Sommer, Tim R. Glowka, Jörg C. Kalff, Tim O. Vilz

**Affiliations:** Klinik- und Poliklinik für Allgemein‑, Viszeral‑, Thorax- und Gefäßchirurgie, Universitätsklinikum Bonn, Venusberg-Campus 1, 53127 Bonn, Deutschland

**Keywords:** Laparoskopie, daVinci®, Rektumresektion, Evidenzbasierte Medizin, Laparoscopy, daVinci®, Rectal resection, Evidence-based medicine

## Abstract

**Hintergrund:**

Robotergestützten Rektumresektionen wird nachgesagt, dass sie bekannte Schwierigkeiten der laparoskopischen Rektumchirurgie durch technische Vorteile überwinden und so zu besseren Behandlungsergebnissen führen. Veröffentlichte Studien berichten jedoch sehr heterogene Ergebnisse. Ziel dieser Arbeit ist es daher, festzustellen, ob es eine Klasse-1a-Evidenz für den Vergleich von robotergestützten vs. laparoskopischen Rektumresektionen gibt. Weiterhin möchten wir die Behandlungsergebnisse unserer Klinik mit den berechneten Effekten aus der Literatur vergleichen.

**Material und Methoden:**

Eine systematische Literaturrecherche nach Klasse-1a-Evidenz wurde durchgeführt und die berechneten Effekte für 7 vorausgewählte Endpunkte wurden miteinander verglichen. Anschließend analysierten wir alle elektiven Rektumresektionen, die zwischen 2017 und 2020 in unserer Klinik durchgeführt wurden, und verglichen die Behandlungsergebnisse mit den Ergebnissen der identifizierten Metaanalysen.

**Ergebnisse:**

Die Ergebnisse der 7 identifizierten Metaanalysen zeigten keine homogenen Effekte für die Endpunkte Operationszeit und Konversionsrate, während die berechneten Effekte der anderen untersuchten Endpunkte weitgehend konsistent waren. Unsere Patientendaten zeigten, dass robotergestützte Rektumresektionen mit signifikant längeren Operationszeiten assoziiert waren, während die anderen Outcomes kaum von der Operationstechnik beeinflusst wurden.

**Diskussion:**

Obwohl bereits Klasse-1a-Metaanalysen zum Vergleich von robotergestützten und laparoskopischen Rektumresektionen vorliegen, erlauben diese keine evidenzbasierte Empfehlung zur Bevorzugung einer der beiden Operationstechniken. Die Analyse unserer Patientendaten zeigte, dass die in unserer Klinik erzielten Ergebnisse weitgehend mit den beobachteten Effekten der Metaanalysen übereinstimmen.

**Zusatzmaterial online:**

Zusätzliche Informationen sind in der Onlineversion dieses Artikels (10.1007/s00104-022-01774-w) enthalten.

## Abstract (en)

**Background:**

Robot-assisted rectal resections are said to overcome the known difficulties of laparoscopic rectal surgery through technical advantages, leading to better treatment results; however, published studies reported very heterogeneous results. The aim of this paper is therefore to determine whether there is class 1a evidence comparing robotic versus laparoscopic rectal resections. Furthermore, we would like to compare the treatment results of our clinic with the calculated effects from the literature.

**Material and methods:**

A systematic literature search for class 1a evidence was performed and the calculated effects for 7 preselected outcomes were compared. We then analyzed all elective rectal resections performed in our hospital between 2017 and 2020 and compared the treatment outcomes with the results of the identified meta-analyses.

**Results:**

The results of the 7 identified meta-analyses did not show homogeneous effects for the outcomes operating time and conversion rate, while the calculated effects of the other outcomes studied were largely consistent. Our patient data showed that robotic rectal resections were associated with significantly longer operation times, while the other outcomes were hardly influenced by the surgical technique.

**Discussion:**

Although class 1a meta-analyses comparing robotic and laparoscopic rectal resections already exist, they do not enable an evidence-based recommendation regarding the preference of one of the two surgical techniques. The analysis of our patient data showed that the results achieved in our clinic are largely consistent with the observed effects of the meta-analyses.

Seit der ersten radikalen Rektumkarzinomresektion durch Miles im Jahr 1907 haben sich die Methoden und technischen Möglichkeiten in der onkologischen Rektumchirurgie kontinuierlich weiterentwickelt. Ein Meilenstein in dieser Entwicklung war die Einführung der totalen mesorektalen Exzision (TME) durch Bill Heald in den frühen 1980er-Jahren. Ein weiterer Meilenstein ist die minimal-invasive Chirurgie, die Anfang der 1990er-Jahre in Form der Laparoskopie eingeführt wurde und derzeit als roboterassistierte Chirurgie weiterentwickelt wird.

## Hintergrund und Fragestellung

**Figure 1:**
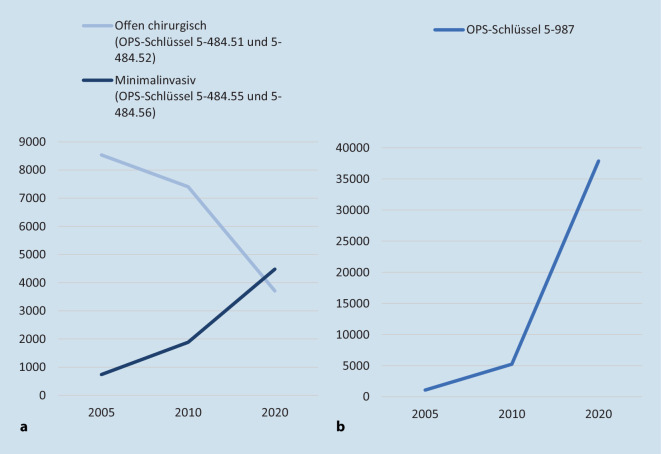

Heutzutage werden elektive kolorektale Resektionen zunehmend laparoskopisch durchgeführt [33]. Die Gründe für den steigenden Anteil laparoskopischer Rektumresektionen liegen in den mittlerweile gut dokumentierten und evidenzbasierten Vorteilen der minimal-invasiven Chirurgie. Dazu gehören beispielsweise ein geringerer Blutverlust, die kürzere Dauer eines postoperativen Ileus, eine niedrigere Inzidenz von Wundinfektionen sowie Narbenhernien, geringere Schmerzen und ein besseres kosmetisches Erscheinungsbild bei gleichwertigen onkologischen Ergebnissen im Vergleich zur offenen Operation ([14, 30, 33]; Abb. 1).

Laparoskopische Rektumresektionen sind jedoch mit einigen Herausforderungen verbunden. Dazu gehören eine flache Lernkurve, ein eingeschränkter Bewegungsumfang aufgrund nicht artikulierbarer Instrumente und eine unnatürliche Hand-Augen-Koordination aufgrund des Verlusts der dreidimensionalen Sicht [14, 33]. Die robotergestützte Chirurgie, als Weiterentwicklung der Laparoskopie, zielt darauf ab, diese Schwierigkeiten mithilfe eines Operationsroboters zu umgehen. So können Robotersysteme mit gelenkigen, hochflexiblen Instrumenten sieben Bewegungsgrade erreichen, während in der Laparoskopie nur vier Bewegungsgrade möglich sind [33]. Dies ermöglicht eine Vielzahl von Zugangswinkeln und sollte eine schärfere und genauere Dissektion entlang des Mesorektums – insbesondere im unteren Drittel des Rektums – ermöglichen, was im Vergleich zur laparoskopischen Rektumchirurgie zu weniger Blutverlust, einer gewebeschonenderen Operation und insbesondere zu einer geringeren Konversionsrate führen soll [12, 33]. Weitere Vorteile des Robotersystems sind die vom Chirurgen gesteuerten Halteinstrumente und die stabile Kamera mit stereoskopischer Sicht, die einen stabilen, dreidimensionalen Blick auf das Operationsfeld ermöglicht. Im Vergleich zur laparoskopischen Chirurgie können so Strukturen wie der Plexus hypogastricus inferior, die Ureteren oder die Gefäßstrukturen leichter identifiziert und präpariert werden, was Komplikationen durch Verletzungen dieser Strukturen verhindern soll [26]. Darüber hinaus ergeben sich einige Vorteile für den Chirurgen, so soll die ergonomischere Haltung beispielsweise zu mehr Komfort und weniger Ermüdung führen [12, 26, 33].

Jedoch gibt es auch Kritikpunkte hinsichtlich des Einsatzes robotischer Verfahren. So werden beispielsweise längere Vorbereitungs- und Operationszeiten häufig als Nachteil robotergestützter Eingriffe angeführt [26, 33]. Ein weiterer Faktor, der vor allem in Zeiten knapper werdender Ressourcen und zunehmender Finanzierungslücken im Gesundheitssystem in den Fokus rückt, sind die hohen Anschaffungs- und Unterhaltskosten [26]. Da in den letzten 10 Jahren nur ein Unternehmen Robotersysteme vertrieb, waren die Preise aufgrund des fehlenden Wettbewerbs exorbitant hoch. In den letzten 3 Jahren haben jedoch mehrere andere Anbieter von Roboterplattformen eine FDA-Zulassung erhalten, was zu einer beginnenden Marktregulierung und einer wettbewerbsgetriebenen Entwicklung neuer Funktionen wie haptisches Feedback oder Eye-Tracking geführt hat [12, 23].

Es existieren bereits zahlreiche Fallserien, Registeranalysen sowie Studien und Übersichtsarbeiten, die die Wirksamkeit und Sicherheit der robotergestützten Rektumchirurgie im Vergleich zur laparoskopischen Chirurgie untersucht haben [3, 19, 20, 26, 27]. Die publizierten Daten sind allerdings divergent und die Qualität der einzelnen Studien unterschiedlich. Dies macht eine objektive Beurteilung der robotergestützten Rektumchirurgie im Vergleich zur laparoskopischen Chirurgie schwierig und das Aussprechen einer evidenzbasierten Empfehlung unmöglich.

Ziel dieser Arbeit war es daher, im Rahmen einer systematischen Literaturrecherche die Klasse-1a-Evidenz (Metaanalyse mehrerer randomisierter kontrollierter Studien [RCTs]) zum Vergleich von robotergestützten vs. laparoskopischen Rektumresektionen zu identifizieren und zu untersuchen, ob zumindest für eine kleine Auswahl von Outcomeparametern homogene Ergebnisse vorliegen und so eine valide Empfehlung ausgesprochen werden kann. Darüber hinaus haben wir in einem zweiten Schritt die Patientendaten unserer Klinik mit den Ergebnissen der Literatur vergleichen, um zu evaluieren, ob die in den Metaanalysen berechneten Effekte mit den von uns in der klinischen Praxis erzielten Behandlungsergebnissen vergleichbar sind.

## Studiendesign und Untersuchungsmethoden

### Scoping-Review

Wir führten eine systematische Literatursuche in Medline (via PubMed) und der Cochrane Library nach systematischen Reviews mit Metaanalysen randomisierter kontrollierter Studien durch, die robotergestützte mit laparoskopischen Rektumresektionen verglichen haben (Suchstrategien siehe Supplement 1).

Alle identifizierten Artikel wurden von zwei unabhängigen Reviewern mithilfe des Onlinetools Rayyan (https://www.rayyan.ai/) gescreent. Im Einzelnen prüften beide Reviewer die Titel und Abstracts der gefundenen Artikel auf ihre Eignung im Hinblick auf die Ein- und Ausschlusskriterien, die in Tab. 1 aufgeführt sind. Entsprechend den Empfehlungen der Cochrane Collaboration zur Durchführung eines systematischen Reviews wurden die Bewertungen verglichen und bei Unstimmigkeiten ein dritter Autor hinzugezogen, um einen Konsens zu erzielen. Anschließend wurden die Volltexte der potenziell relevanten Metaanalysen von den beiden Reviewern unabhängig voneinander gesichtet. Auch hier wurden Meinungsverschiedenheiten über den Einschluss der Reviews durch Bewertung eines dritten Autors behoben.

**Table 1:** 

	Einschlusskriterien	Ausschlusskriterien
Untersuchte Patientenpopulation	Studien, die Patienten einschlossen, die sich einer elektiven Rektumresektion unterzogen, unabhängig von der Operationsindikation	Einschluss von Studien, die nicht auf elektive Rektumresektionen beschränkt waren
Untersuchter Vergleich	Robotergestützte vs. laparoskopische Eingriffe, unabhängig von dem verwendeten robotergestützten oder laparoskopischen Operationssystem	Metaanalysen, die robotergestützte und laparoskopische Eingriffe nicht miteinander, sondern mit offenen Eingriffen vergleichen, waren nicht zugelassen. Auch der Vergleich verschiedener Roboter- oder laparoskopischer Systeme untereinander war nicht zulässig
Publikationstyp	Systematischer Review mit Metaanalyse von RCTs	Reviews, die keine systematische Literaturrecherche durchgeführt haben oder keine Zusammenfassung der Ergebnisse in Form einer Metaanalyse enthalten. Ebenfalls ausgeschlossen wurden Metaanalysen, die neben RCTs auch nichtrandomisierte Studien einschlossen

*RCTs* randomisierte kontrollierte Studien

Anschließend wurde die methodische Qualität der eingeschlossenen Übersichtsarbeiten gemäß den Empfehlungen von Cochrane Deutschland und der Arbeitsgemeinschaft der Wissenschaftlichen Medizinischen Fachgesellschaften (AWMF) zur Evidenzaufbereitung anhand der AMSTAR-2-Kriterien bewertet [4, 25]. Um die Ergebnisse der eingeschlossenen Metaanalysen vergleichen zu können, wurden die berechneten Effektschätzer mit 95 %-Konfidenzintervallen (KI) und *p*-Wert-Signifikanztest für den Gesamteffekt für die folgenden Endpunkte unabhängig von zwei Reviewern extrahiert:

- Operationsdauer,
- Konversion zum offenen Eingriff,
- postoperative Gesamtkomplikationen,
- Anastomoseninsuffizienzen,
- Mortalität,
- Zeit bis zum ersten Stuhlgang,
- Dauer des Krankenhausaufenthalts.

### Retrospektive Analyse eigener Patientendaten

Alle elektiven Rektumresektionen (ausgenommen wurden Rektumexstirpationen und Proktokolektomien), die im Zeitraum vom 01.01.2017 bis zum 31.12.2020 in der Klinik und Poliklinik für Allgemein‑, Viszeral‑, Thorax- und Gefäßchirurgie des Universitätsklinikums Bonn durchgeführt wurden, wurden retrospektiv ausgewertet. Neben den Patientencharakteristika wurden Daten zu den oben genannten Outcomes erhoben, um die postoperativen Ergebnisse unserer Klinik mit denen der Literatur zu vergleichen.

Die statistische Analyse wurde mit Microsoft Excel und RevMan5 durchgeführt. Dichotome Ergebnisse werden als Risikoverhältnis (Risk Ratio, RR) mit 95 %-KI angegeben, kontinuierliche Daten als mittlere Differenz („mean difference“, MD) mit 95 %-KI.

## Ergebnisse

### Suche und Identifikation von Level-1a-Evidenz

**Figure 2:**
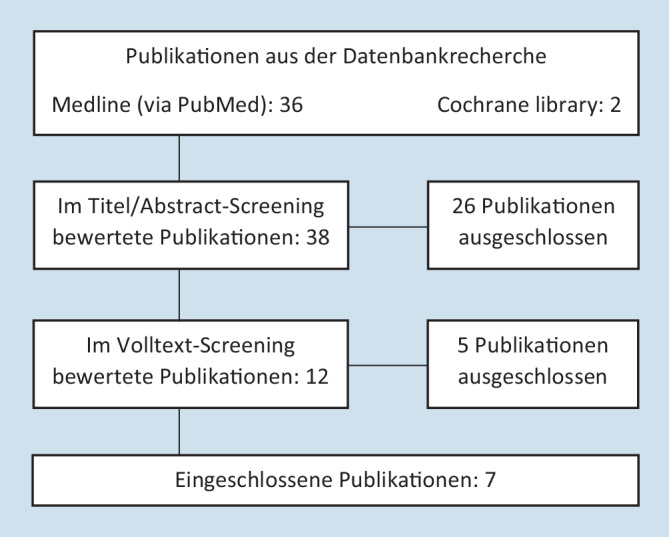

Bei den Datenbankrecherchen wurden insgesamt 38 potenziell infrage kommende Reviews gefunden. Davon wurden 26 im Rahmen des Titel‑/Abstract-Screenings ausgeschlossen. Von den verbleibenden 12 Artikeln wurden 5 bei der Volltextauswertung ausgeschlossen, da sie die Einschlusskriterien nicht erfüllten (alle im Rahmen des Volltextscreenings ausgeschlossenen Reviews schlossen sowohl randomisierte als auch nichtrandomisierte Studien in ihre Metaanalysen ein). Schließlich wurden 7 Klasse-1a-Publikationen identifiziert, die robotergestützte mit laparoskopischen Rektumresektionen verglichen haben. Das Flussdiagramm in Abb. 2 veranschaulicht den Auswahlprozess.

### Methodische Bewertung und Zusammenfassung der Reviewergebnisse

Die Bewertung der eingeschlossenen Reviews nach AMSTAR 2 (Zitat) ergab Werte zwischen 8,5 und 12 von 16. Die Bewertungen der einzelnen Reviews sind in Tab. 2 aufgeführt. Abzüge in der AMSTAR-Bewertung gab es unter anderem wegen fehlender Protokolle mit vorgegebener Methodik, unzureichend berichteter Suchstrategien, der fehlenden Angabe von Ausschlussgründen im Volltextscreening oder der unzureichenden Diskussion des Einflusses von „risk of bias“ oder Heterogenität auf die Ergebnisse.

Die älteste der eingeschlossenen Metaanalysen wurde 2014 veröffentlicht und basierte auf 4 RCTs [16]. Die jüngste stammt aus dem Frühjahr 2021 und basiert auf 7 RCTs [28]. Mit 9 eingeschlossenen RCTs ist die Metaanalyse von Eltair et al. aus dem Jahr 2020 die Übersichtsarbeit mit den meisten Studien [8]. Insgesamt gibt es jedoch eine große Überschneidung der eingeschlossenen Studien (Tab. 2).

**Table 2:** 

Review	Eingeschlossene Studien	AMSTAR-Bewertung
Tang 2021 [28]	7 RCTs [1, 5, 11, 15, 22, 27, 31]	9,5 von 16
Eltair 2020 [8]	9 RCTs [1, 5, 11, 13, 15, 22, 29, 31, 34]	11 von 16
Han 2020 [10]	8 RCTs [1, 5, 11, 13, 15, 22, 29, 31]	11 von 16
Liao 2019 [17]	7 RCTs [1, 5, 11, 13, 15, 22, 31]	10,5 von 16
Li 2019 [18]	7 RCTs [1, 5, 11, 15, 22, 29, 31]	8,5 von 16
Prete 2018 [24]	5 RCTs [1, 11, 13, 22, 31]	12 von 16
Liao 2014 [16]	4 RCTs [1, 13, 21, 22]	10 von 16

Im Folgenden werden die Ergebnisse der Metaanalysen zu den oben genannten Outcomeparametern berichtet:

#### Operationsdauer.

Dieser Endpunkt wurde in 5 der einbezogenen Reviews bewertet. Drei von diesen berichten, dass die robotergestützte Chirurgie in einer längeren Operationszeit resultiert, während zwei keinen Unterschied zwischen robotergestützten und laparoskopischen Eingriffen fanden (Tab. 3).

**Table 3:** 

Review		Effektschätzer
Eltair 2020 [8]	Längere Operationszeiten bei robotergestützten Eingriffen	MD 31,64 (95 %-KI 12,09 bis 51,19)
Han 2020 [10]		MD 33,28 (95 %-KI 8,92 bis 57,65)
Prete 2018 [24]		MD 38,43 (95 %-KI 31,84 bis 45,01)
Li 2019 [18]	Kein Unterschied der Operationszeiten	MD 27,04 (95 %-KI −1,06 bis 55,14)
Liao 2014 [16]		MD 23,89 (95 %-KI 12,09 bis 59,87)

#### Konversion zum offenen Eingriff.

Dieser Endpunkt wurde in 6 der eingeschlossenen Reviews untersucht. Vier Metaanalysen berichten, dass die robotergestützte Chirurgie zu einer niedrigeren Konversionsrate führt, während zwei keinen Unterschied zwischen robotergestützten und laparoskopischen Eingriffen feststellen konnten (Tab. 4).

**Table 4:** 

Review		Effektschätzer
Han 2020 [10]	Niedrigere Konversionsraten bei robotergestützten Eingriffen	RR 0,45 (95 %-KI 0,21 bis 0,94)
Li 2019 [18]		OR 0,29 (95 %-KI 0,09 bis 0,96)
Prete 2018 [24]		RR 0,58 (95 %-KI 0,35 bis 0,97)
Liao 2014 [16]		OR 0,25 (95 %-KI 0,07 bis 0,91)
Tang 2021 [28]	Kein Unterschied der Konversionsrate	OR 0,61 (95 %-KI 0,35 bis 1,07)
Eltair 2020 [8]		RR 0,46 (95 %-KI 0,18 bis 1,01)

#### Postoperative Gesamtkomplikationen, Anastomoseninsuffizienzen und Mortalität.

Das Auftreten postoperativer Komplikationen wurde in 5 Reviews, von Anastomoseninsuffizienzen in 4 Reviews und die Mortalität in 2 Reviews untersucht. In keiner der berechneten Metaanalysen konnte ein Einfluss des chirurgischen Zugangsweges auf die genannten Endpunkte nachgewiesen werden.

#### Zeit bis zum ersten Stuhlgang.

Dieser Endpunkt wurde in 4 der eingeschlossenen Studien untersucht. Alle zeigten eine Verkürzung der Zeit bis zum ersten Stuhlgang, wobei dieser Effekt nur in 2 der 4 Studien statistisch signifikant war (Tab. 5).

**Table 5:** 

Review		Effektschätzer
Han 2020 [10]	Früherer erster Stuhlgang nach robotergestützten Eingriffen	MD −0,29 (95 %-KI −0,66 bis 0,08)
Li 2019 [18]		MD −0,06 (95 %-KI −0,35 bis 0,22)
Prete 2018 [24]		MD −0,59 (95 %-KI −0,95 bis −0,23)
Liao 2014 [16]		MD −0,54 (95 %-KI −0,93 bis −0,14)

#### Krankenhausverweildauer.

Dieser Endpunkt wurde in 5 der eingeschlossenen Reviews untersucht. Während in 4 davon kein Einfluss des chirurgischen Verfahrens auf die Dauer des Krankenhausaufenthalts gefunden wurde, berichtete eine Übersichtsarbeit über eine kürzere Krankenhausverweildauer nach robotergestützten Eingriffen (Tab. 6).

**Table 6:** 

Review		Effektschätzer
Eltair 2020 [8]	Kein Unterschied der Krankenhausverweildauer	MD −0,60 (95 %-KI −1,36 bis 0,16)
Han 2020 [10]		MD −0,51 (95 %-KI −1,53 bis 0,50)
Li 2019 [18]		MD −0,51 (95 %-KI −1,92 bis 0,90)
Prete 2018 [24]		MD −0,61 (95 %-KI −2,23 bis 1,02)
Liao 2014 [16]		MD −0,53 (95 %-KI −2,14 bis 2,08)

### Ergebnisse der retrospektiven Analyse klinikinterner Daten

Während des Auswertungszeitraums wurden insgesamt 51 elektive, minimal-invasive Rektumresektionen durchgeführt (35 robotisch [daVinci XI] und 16 laparoskopisch). Zwischen den beiden Gruppen gab es keine signifikanten Unterschiede in Bezug auf das Geschlecht (*p* = 0,99), das Alter (*p* = 0,68), das Körpergewicht (*p* = 0,22) oder die ASA-Klassifizierung der Patienten (*p* = 0,78).

Die Ergebnisse der Analyse unserer Patientendaten im Hinblick auf die oben genannten Outcomeparameter sind in Tab. 7 aufgeführt. Da keiner der 51 eingeschlossenen Patienten während seines Krankenhausaufenthaltes verstarb, konnte für diesen Endpunkt keine Risikoreduktion berechnet werden.

**Table 7:** 

Outcome Parameter	Effektschätzer
Operationsdauer	MD 153,45 (95 %-KI 98,94 bis 207,96)
Konversion zum offenen Eingriff	RR 0,69 (95 %-KI 0,13 bis 3,71)
Postoperative Gesamtkomplikationen	RR 1,37 (95 %-KI 0,43 bis 4,40)
Anastomoseninsuffizienzen	RR 0,42 (95 %-KI 0,02 bis 8,35)
Zeit bis zum ersten Stuhlgang	MD 0,39 (95 %-KI −0,54 bis 1,32)
Dauer des Krankenhausverweildauer	MD −0,93 (95 %-KI −6,38 bis 4,52)

## Diskussion

### Vergleich der internen Daten mit den Ergebnissen der Metaanalysen

Die Ergebnisse der Auswertung der im Beobachtungszeitraum am Universitätsklinikum Bonn durchgeführten Rektumresektionen stimmen überwiegend mit den Erkenntnissen der Metaanalysen überein. Sowohl hinsichtlich des Auftretens postoperativer Komplikationen im Allgemeinen als auch hinsichtlich des Auftretens von Anastomoseninsuffizienzen zeigte die Auswertung unserer Patienten keinen Unterschied zwischen robotergestützten und laparoskopischen Eingriffen, analog zu den Ergebnissen der Metaanalysen. Auch die Krankenhausverweildauer wurde in unserem Kollektiv nicht durch die Operationstechnik beeinflusst, was ebenfalls den Ergebnissen der 5 Metaanalysen, die diesen Endpunkt untersuchten, entspricht.

Bezüglich des Auftretens des ersten postoperativen Stuhlgangs berichten die Metaanalysen zwar eine geringgradige Reduktion, da es sich aber um eine Reduktion von weniger als einem Tag handelt, hat dieser Unterschied keine klinische Relevanz. Daher muss auch hier von einem fehlenden Effekt ausgegangen werden muss. Auch unsere Daten konnten keinen Einfluss der Operationstechnik auf das Auftreten des ersten Stuhlgangs zeigen.

Abweichungen zwischen den Ergebnissen der Metaanalysen und unseren eigenen Daten gibt es jedoch bei den Endpunkten „Dauer der Operation“ und „Konversionsrate“. Während es innerhalb der Metaanalysen keinen Konsens über den Effekt der robotergestützten Chirurgie auf die Operationsdauer gibt, zeigt unsere Auswertung einen deutlichen Unterschied. So dauerten robotergestützte Rektumresektionen am Universitätsklinikum Bonn im Durchschnitt 2,5 h länger als laparoskopische. Allerdings korreliert die Operationsdauer in hohem Maße mit der Expertise beim jeweiligen Verfahren. In diesem Falle war es so, dass die Lernkurve der drei Robotikoperateure genau in den untersuchten Zeitraum fällt, wohingegen alle drei eine hohe Expertise im Bereich der Laparoskopie hatten und diese Eingriffe seit Jahren routinemäßig durchführen. Daher könnte man zumindest eine moderate Angleichung der Operationszeit durch mehr Erfahrung mit dem robotergestützten System erwarten.

In Bezug auf die Konversionsrate zeigen die Reviews ebenfalls kein einheitliches Ergebnis. Während 4 der 6 Metaanalysen eine Reduktion der Konversionsrate nachweisen konnten, zeigte unsere Analyse keinen statistisch signifikanten Unterschied zwischen robotergestützten und laparoskopischen Eingriffen. Jedoch stimmt ein Trendunterschied von 12,5 % (2 Konversionen bei 16 laparoskopischen Eingriffen) gegenüber 8,6 % (3 Konversionen von 35 robotergestützten Eingriffen) mit den Daten der Metaanalysen überein. Aufgrund der geringen Fallzahl unserer Analyse sind diese Daten jedoch wenig verlässlich.

### Beurteilung der verfügbaren Klasse-1a-Evidenz

Unser Scoping-Review konnte zeigen, dass es bereits Klasse-1a-Evidenz zum Vergleich von robotergestützten vs. laparoskopischen Rektumresektionen gibt [8, 10, 16–18, 24, 28]. Die identifizierten Reviews unterscheiden sich jedoch nicht nur hinsichtlich der Auswahl der untersuchten Outcomeparameter, sondern auch in Bezug auf die methodische Qualität und die berechneten Effektschätzer einzelner Outcomes.

Zu den methodischen Einschränkungen gehören Kritikpunkte wie das Fehlen eines Protokolls mit vorab festgelegter Methodik sowie fehlende oder unzureichend durchgeführte Untergruppen- und Sensitivitätsanalysen. Obwohl die meisten Reviews das Biasrisiko der einzelnen Studien bewerten, wurde diese Bewertung bei der Interpretation der Ergebnisse meist nicht berücksichtigt. Darüber hinaus wird zwar in jeder Metaanalyse die Heterogenität zwischen den eingeschlossenen Studien berechnet und bei vorhandener Heterogenität teilweise sogar als limitierender Faktor der Analyse genannt, aber es wird selten versucht, die Gründe für die Heterogenität herauszufinden.

Um das Ergebnis einer Metaanalyse besser bewerten zu können, sollten neben dem berechneten Effekt auch andere Aspekte wie das Biasrisiko der eingeschlossenen Studien oder eine nachgewiesene Heterogenität zwischen den Studien in die Interpretation der Ergebnisse einbezogen werden. Der GRADE-Ansatz ist eine inzwischen gut bekannte Methode, die darauf abzielt, das Vertrauen in die Evidenz anhand von 8 Punkten zu prüfen [9]. Keine der identifizierten Metaanalysen hat diesen oder einen ähnlichen Ansatz zur Interpretation ihrer Ergebnisse verwendet, was es dem Leser unmöglich macht, das Vertrauen in die Evidenz zu beurteilen.

Die Gründe für die abweichenden Ergebnisse liegen vor allem in der Variation der eingeschlossenen Studien. Neben dem Aspekt der zeitlichen Verfügbarkeit – in älteren Metaanalysen fehlen neuere RCTs – ergeben sich die Unterschiede aus Abweichungen bei der Literatursuche und Studienauswahl. So enthält der Review von Liao 2014 [16] auch eine randomisierte klinische Studie zu roboterassistierten vs. laparoskopischen rechtsseitigen Hemikolektomien. Aber auch andere eingeschlossene Metaanalysen zeigen Abweichungen von der in ihrer Methodik definierten Population. Mit Ausnahme der Metaanalyse von Liao 2014 [16] geben alle Reviews an, dass die zu untersuchende Population auf Patienten mit Rektumkarzinom beschränkt war. Dennoch ist die 2011 publizierte RCT von Rodriguez et al. [13] in den Metaanalysen von Eltair 2020 [8], Han 2020 [10] und Prete 2018 [24] enthalten, obwohl diese Studie hauptsächlich Sigmaresektionen untersuchte.

Insgesamt beruhen die Ergebnisse der hier betrachteten Metaanalysen auf 11 verschiedenen RCTs [1, 5, 11, 13, 15, 21, 22, 27, 29, 31, 34], wobei die eingeschlossenen Studien je Review stark variieren, obwohl die genannten Ein- und Ausschlusskriterien weitgehend identisch sind. Eine Auflistung der im Volltextscreening ausgeschlossenen Studien mit den jeweiligen Ausschlussgründen könnte Aufschluss darüber geben, ob die gefundenen Unterschiede auf der durchgeführten Suche oder dem erfolgten Auswahlprozess beruhen. Diese Information wurde jedoch in keiner der gefundenen Reviews berichtet.

Zusammenfassend ist festzuhalten, dass die Frage der Effektivität und Sicherheit der robotergestützten Rektumchirurgie im Vergleich zu laparoskopischen Verfahren noch nicht abschließend geklärt ist, obwohl Evidenz der Klasse 1a verfügbar ist. Weitere Metaanalysen zu diesem Thema sollten klar definierte Ein- und Ausschlusskriterien enthalten, die bereits in einem Protokoll festgelegt sind und den Prozess der Literatursuche und -auswahl möglichst detailliert und nachvollziehbar beschreiben. Darüber hinaus ist eine adäquate Qualitätsbewertung der eingeschlossenen Studien erforderlich, deren Ergebnisse ebenfalls in die Bewertung der Ergebnisse einfließen. Zusätzlich sollten die Ergebnisse auch auf ihre Verlässlichkeit hin überprüft werden, bestenfalls anhand des GRADE-Ansatzes.

Abschließend gilt es jedoch zu beachten, dass Metaanalysen nur so gut sein können wie die eingeschlossenen Studien. Die bisher veröffentlichten und in die untersuchten Metaanalysen inkludierten RCTs weisen einige Einschränkungen auf (z. B. geringer Stichprobenumfang, breite Population ohne Subgruppenanalysen, methodische Limitationen etc.). Um die Effektivität der robotergestützten Chirurgie bei Rektumkarzinomen besser beurteilen zu können, ist es daher notwendig, weitere qualitativ hochwertige RCTs durchzuführen und mehr Evidenz zu generieren, die in zukünftigen Metaanalysen zu einer besseren Beurteilbarkeit beitragen kann.

Eine Einschränkung aller Metaanalysen zu diesem Vergleich ist jedoch, dass RCTs in der Regel nur an kolorektalen Zentren oder Kliniken mit hohen Fallzahlen und Expertise sowohl in der robotergestützten als auch in der laparoskopischen Rektumchirurgie durchgeführt werden. Angesichts des bekannten Volume-Outcome-Effekts [2, 6, 7] ist daher zu erwarten, dass in diesen Studien bessere Ergebnisse erzielt werden als in kleineren Kliniken mit geringerer Expertise, sodass die Ergebnisse dieser Studien sowie der darauf basierenden Metaanalysen hinsichtlich ihrer Übertragbarkeit in den klinischen Alltag immer kritisch hinterfragt werden müssen, insbesondere, weil die robotische Chirurgie durch ihre relative Neuartigkeit noch nicht so weit verbreitet ist wie die konventionelle Laparoskopie.

### Limitationen unserer Überprüfung

Eine der Einschränkungen unserer Auswertung besteht darin, dass die Literatursuche auf nur zwei Datenbanken beschränkt war. Aufgrund einer großen Überschneidung zwischen Medline und Embase sowie einer fehlenden Lizenz wurde eine Suche in Embase für diese Überprüfung nicht durchgeführt. Eine zusätzliche Suche in Registern für systematische Übersichten wie PROSPERO (https://www.crd.york.ac.uk/PROSPERO/) hätte jedoch weitere Metaanalysen sowie geplante Projekte aufzeigen können.

Eine weitere Einschränkung besteht darin, dass die in dieser Übersichtsstudie bewerteten klinischen Ergebnisse nur einen Bruchteil der für die Entscheidung relevanten Endpunkte, welches chirurgische Verfahren bevorzugt werden sollte, untersucht. Insbesondere pathoonkologische Aspekte wie die Vollständigkeit des Resektats oder patientenrelevante Aspekte wie die Häufigkeit postoperativer Harn- oder Sexualfunktionsstörungen wurden in dieser Auswertung nicht berücksichtigt. Die Deutsche Gesellschaft für Allgemein- und Viszeralchirurgie (DGAV) hat bereits 2017 Qualitätsindikatoren für die Rektumkarzinomchirurgie definiert. Dazu gehören ein tumorfreier zirkumferenzieller Resektionsrand, die Rate an Anastomoseninsuffizienzen und abdominellen Wundheilungsstörungen, die Rate an Patienten mit einer neu angelegten permanenten Harnableitung und der damals neu definierte Endpunkt MTL30, der das Versterben des Patienten innerhalb von 30 Tagen nach der Operation, die Verlegung in ein anderes Akutkrankenhaus innerhalb von 30 Tagen oder einen stationären Aufenthalt von mehr als 30 Tagen subsumiert. Von diesen Qualitätsindikatoren haben wir allerdings nur Anastomoseninsuffizienzen und Teilaspekte von MTL30 untersucht [32].

## Fazit für die Praxis

- Es gibt bereits Evidenz der Klasse 1a für den Vergleich von robotergestützten und laparoskopischen Rektumresektionen, aber die Aussagen sind nicht eindeutig.
- Zukünftige Metaanalysen zu diesem Thema sollten auf eine nachvollziehbare Studienauswahl achten und Überlegungen zur Zuverlässigkeit der Evidenz in die Interpretation der Ergebnisse einbeziehen.
- Die in unserer Klinik erzielten Behandlungsergebnisse stimmen überwiegend mit den in den Metaanalysen beschriebenen Effekten überein.

## Supplementary Information




