# Supplementary material for: Robot-assisted rectal resections—Scoping review for level 1a evidence and retrospective analysis of in-clinic data
Source: Chirurgie (Heidelb). 2022 Nov 30;94(2):138–46. [Article in German] doi: 10.1007/s00104-022-01774-w (PMC9898418; doi:10.1007/s00104-022-01774-w)
Supplement: Supplementary file 1 [file 104_2022_1774_MOESM1_ESM.docx]

**Suchstrategie PubMed**

#1 "Colorectal Surgery"[Majr] OR "Proctectomy "[Mesh] OR "Rectum/surgery"[Mesh]

#2 rectum [tiab] OR rectal [tiab] OR proctolog* [tiab] OR proctocolonic [tiab]

#3 surger* [tiab] OR surgical* [tiab] OR resect* [tiab] OR incisi* [tiab] OR excisi* [tiab] OR invasive* [tiab] OR restorati* [tiab] OR operation* [tiab] OR operative* [tiab] OR perioperati* [tiab] OR peri-operati* [tiab] OR "surgery"[Subheading]

#4 #2 AND #3

#5 #1 OR #4

#6 "Robotic Surgical Procedures"[Mesh] OR "Robotics"[Majr]

#7 robotic [tiab] OR robot [tiab] OR robot-assisted [tiab] OR robot assisted [tiab] OR robotic-assisted [tiab] OR robotic assisted [tiab] OR robotics [tiab] OR robotised [tiab] OR da vinci [tiab]

#8 #7 OR #6

#9 #5 AND #8

#10 meta-analysis [Filter] OR systematicreview [Filter] OR meta analysis [tiab] OR meta-analysis [tiab] OR metaanalysis OR "Meta-Analysis as Topic"[Mesh]

#11 “randomized controlled trial*” [tiab] OR RCT [tiab] OR “randomised controlled trial*” [tiab]

#12 #10 AND #11 AND #9

**Suchstrategie Central**

#1 [mh "colorectal surgery"] OR [mh "colectomy"] OR [mh "proctectomy"]

#2 (rectum OR rectal OR proctolog* OR proctocolonic):ti,ab,kw

#3 (surger* OR surgical* OR resect* OR incisi* OR excisi* OR invasive* OR restorati* OR operation* OR operative* OR perioperati* OR peri-operati*):ti,ab,kw

#4 #2 AND #3

#5 #1 OR #4

#6 [mh "Robotic Surgical Procedures"] OR [mh "Robotics"]

#7 (robotic OR robot OR robot-assisted OR robot assisted OR robotic-assisted OR robotic assisted OR robotics OR robotised OR da vinci):ti,ab,kw

#8 #7 OR #6

#9 #5 AND #8
